# Rapid Remodeling of the Human Gut Microbiome in Response to Short‐Term Animal Product Restriction and Associations with Host Molecular Phenotypes

**DOI:** 10.1002/advs.202515575

**Published:** 2026-02-24

**Authors:** Christina Emmanouil, Maria Anezaki, Alexandros Simistiras, Stavros Glentis, Nikolaos Scarmeas, Pantelis Hatzis, Konstantinos Rouskas, Antigone S. Dimas

**Affiliations:** ^1^ Institute for Bioinnovation Biomedical Sciences Research Center ʻAlexander Fleming’ Vari Greece; ^2^ Pediatric Hematology/Oncology Unit (POHemU), First Department of Pediatrics University of Athens Aghia Sophia Children's Hospital Athens Greece; ^3^ First Department of Neurology, Aiginition Hospital National and Kapodistrian University of Athens Athens Greece; ^4^ Department of Neurology Columbia University New York USA; ^5^ Institute for Fundamental Biomedical Research Biomedical Sciences Research Center ʻAlexander Fleming’ Vari Greece; ^6^ Institute of Applied Biosciences Centre For Research & Technology Hellas Thessaloniki Greece; ^7^ Institute of Translational Genomics Helmholtz Zentrum München – German Research Center for Environmental Health Neuherberg Germany

**Keywords:** animal product restriction, compensatory microbial response, dietary restriction, human health, human gut microbiome, multi‐omics integration, rapid adaptive plasticity

## Abstract

Diet strongly influences the gut microbiome, which in turn influences health, yet the effects of dietary patterns on microbiome composition and function in humans remain underexplored. A unique group of apparently healthy individuals from Greece, who alternate between omnivory and restriction of animal products for religious reasons (periodically restricted group, *n* = 200), has been profiled. Using 16S rRNA sequencing, plasma metabolomics, and proteomics, the impact of three‐to‐four weeks of dietary restriction on gut microbiome composition and function is assessed and associations with host plasma biology are explored. Findings are compared to a continuously omnivorous group profiled in parallel (non‐restricted group, *n* = 211). Animal product restriction reduced microbial diversity, primarily affecting rare taxa, and altered the abundance of nearly one‐third of bacterial genera. Inferred functional shifts included downregulation of pathways contributing to cholesterol biosynthesis and purine degradation, alongside upregulation of vitamin B2 and tryptophan biosynthesis, suggesting compensatory microbial responses to dietary nutrient depletion. Multi‐omics integration revealed four microbial‐metabolite‐protein clusters, including a diet‐responsive module associating *Negativibacillus* with metabolic regulator FGF21 and intermediate‐density lipoproteins. These findings demonstrate rapid adaptive plasticity of the human gut microbiome in response to short‐term dietary restriction and highlight candidate microbial and molecular pathways associated with animal product restriction and host biology.

## Graphical abstract

A real‐world model of structured animal product restriction practiced for religious reasons reveals the dynamic adaptability of the human gut microbiome to dietary change and uncovers reductions in diversity and rare taxa loss. Integrated microbiome, metabolomic, and proteomic analyses uncover coordinated taxonomic and molecular shifts and identify microbial‐metabolite‐protein association clusters, providing insight into dietary restriction, microbiome dynamics, and host biology.

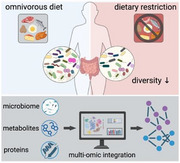

## 1 Introduction

The co‐evolution of mammals and their microbial communities has given rise to complex symbiotic relationships defined by the bidirectional exchange of resources essential for host development, metabolism, and immune regulation [1]. In humans, the gut microbiome plays a central role in maintaining health, contributes to disease development [2, 3], and is strongly shaped by dietary intake [4, 5]. Through the fermentation of proteins and indigestible plant polysaccharides, gut microbes produce a broad spectrum of metabolites, including essential vitamins, amino acids, and short‐chain fatty acids (SCFAs), that influence inflammation, immune responses, energy homeostasis, and neurotransmission [1, 6]. Disruptions in microbial composition and function have been associated with a range of diseases, including obesity, type 2 diabetes, cardiovascular disease, neurodegenerative disorders, and cancer [2, 3, 7, 8].

Dietary interventions are increasingly recognized as effective strategies for the prevention and management of chronic diseases, in part through their impact on the gut microbiome. However, investigating the effects of diet on the microbiome and human health remains challenging due to the complexity of dietary behaviors and interindividual variability [9]. Most dietary studies focusing on the gut microbiome in humans to date are observational, while interventional studies typically involve small sample sizes. Despite these challenges, it is well‐established that diets rich in animal products, particularly red meat, are characterized by increased protein fermentation and enrichment of bile‐metabolizing bacteria such as *Alistipes putredinis*, *Bilophila wadsworthia* and *Ruminococcus torques* [4, 10]. These compositional shifts are often linked to the production of inflammatory metabolites, including trimethylamine N‐oxide (TMAO), and have been implicated in adverse cardiometabolic outcomes [8, 11]. Moreover, Western dietary patterns are characterized by deliberate efforts to increase protein intake. A plausible mechanistic link has been proposed between increased colorectal cancer risk associated with such diets, and the excess protein reaching the colon where microbial fermentation can generate potentially harmful metabolites and promote colonic inflammation [12].

Plant‐based diets, typically rich in fiber, polyphenols, and complex carbohydrates, have been associated with reduced oxidative stress, reduced levels of low‐grade inflammation, and improved metabolic health [13]. These dietary patterns are known to promote the enrichment of polysaccharide‐fermenting and SCFA‐producing gut bacteria, such as *Butyricicoccus* sp. and *Roseburia hominis* [10, 13], microbial shifts generally linked to favorable health outcomes, such as reduced inflammation, lower risk of obesity, type 2 diabetes, cardiovascular disease, and certain cancers [5, 14, 15]. However, plant‐based dietary patterns have also been associated with lower intake of certain micronutrients, particularly vitamins B2, B12, and D, as well as calcium, and with potential health risks such as increased stroke incidence and lower bone mineral density [14, 15].

In the present study we performed 16S gut microbiome profiling of a unique group of apparently healthy individuals who voluntarily alternate between periods of omnivory and dietary restriction of animal products for religious reasons. The consistent nature of this dietary pattern, characterized by abstinence from animal products for 180–200 days annually and maintained in a highly structured manner for over a decade, provides a unique, real‐world model that closely approximates a controlled dietary intervention. Furthermore, religiously motivated adherence overcomes common limitations of dietary intervention studies, including inconsistent intake and poor long‐term compliance, and better reflects sustained, real‐life dietary behavior and its relationship to health. Microbiome profiles were compared between dietary states, and with profiles obtained in parallel from a control group of continuously omnivorous individuals. We uncovered dynamic microbiome responses to short‐term animal product restriction, characterized by a reduction in microbial diversity, and by shifts in microbial abundance and predicted metabolic activity. Building on these findings, the extensive molecular characterization of our cohort enabled interpretation of microbiome changes within a well‐defined host molecular context. By integrating microbiome data with host metabolomic and proteomic profiles, we place diet‐responsive microbiome variation within broader molecular pathways with established relevance to human physiology and health. To our knowledge, this is the first study to integrate microbiome, metabolome, and proteome data within a structured dietary pattern framework.

## 2 Results

### 2.1 Population Sample

**FIGURE 1:**
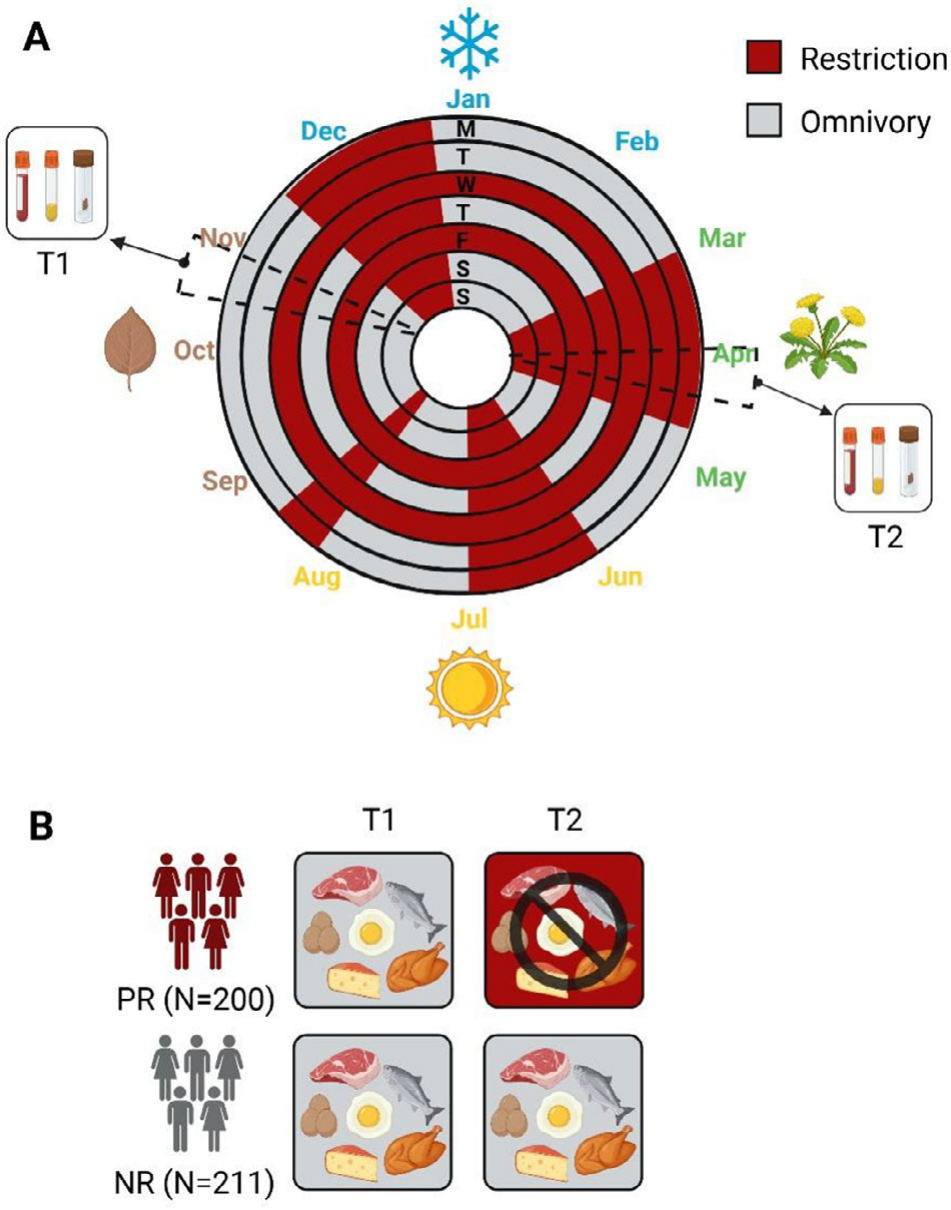
Periods of animal product restriction and study design. (A) Periodically restricted (PR) individuals alternate between omnivory and animal product restriction for religious reasons. Animal product restriction is practiced for 180–200 days annually during four extended periods throughout the year and on Wednesdays and Fridays of each week. (B) FastBio study design. Participants were profiled at T1 (in autumn) and T2 (in spring). Non‐restricted (NR) continuously omnivorous individuals were profiled in parallel, as a control group. Created in BioRender. Emmanouil, C. (2026) https://BioRender.com/k85f93a.

We profiled a unique group of apparently healthy individuals from Greece who alternate between omnivory and dietary restriction of animal products as part of religious fasting. Periodically restricted individuals (PR, *n* = 200, age: 20–76 years, sex: 54% female, BMI: 28.4±4.6 Kg m^−2^, Table S1) abstain from meat, fish, dairy products, and eggs for a total of 180–200 days annually. Abstinence is practiced during four extended periods throughout the year, as well as on Wednesdays and Fridays of each week (Figure 1A). We compared findings to a non‐restricted control group of continuously omnivorous individuals (NR, *n* = 211, age: 19–74 years, sex: 55% female, BMI: 26.2±4.4 Kg m^−2^, Figure 1B; Table S1) profiled in parallel. Participants were profiled at two time points: T1 in autumn, during a period of omnivory for both dietary groups, and T2 in early spring, following three‐to‐four weeks of animal product restriction for PR individuals, during Lent. Our study design therefore involves four dietary group by time point combinations (contexts). Importantly, our study did not include participants who had received antimicrobial medication within six months of T1 (Text S1) and information on antimicrobial use was recorded for participants who had received this type of medication between time points (Text S2).

During Lent, in addition to a pronounced reduction of animal fat intake, PR individuals undergo restriction of protein intake (as a proportion of total energy intake) [16, 17]. This is driven chiefly by abstinence from all sources of animal protein and is not accompanied by changes in total energy or fiber intake [16, 17].We have previously shown that this type of short‐term dietary restriction induces broad immunometabolic reprogramming, with mostly positive effects on health, marked by reductions in plasma levels of cholesterol and other lipid classes, of branched‐chain amino acids, and shifts in immunometabolic regulators, including a pronounced increase in potent metabolic regulator FGF21 [18, 19]. In the present study we explored the effects of this dietary pattern on the gut microbiome using 16S rRNA gene sequencing and combined with findings from plasma biomarkers, metabolites and proteins from the same individuals [18, 19].

### 2.2 Taxonomic Profiling and Diet‐Responsive Shifts in Bacterial Diversity

**FIGURE 2:**
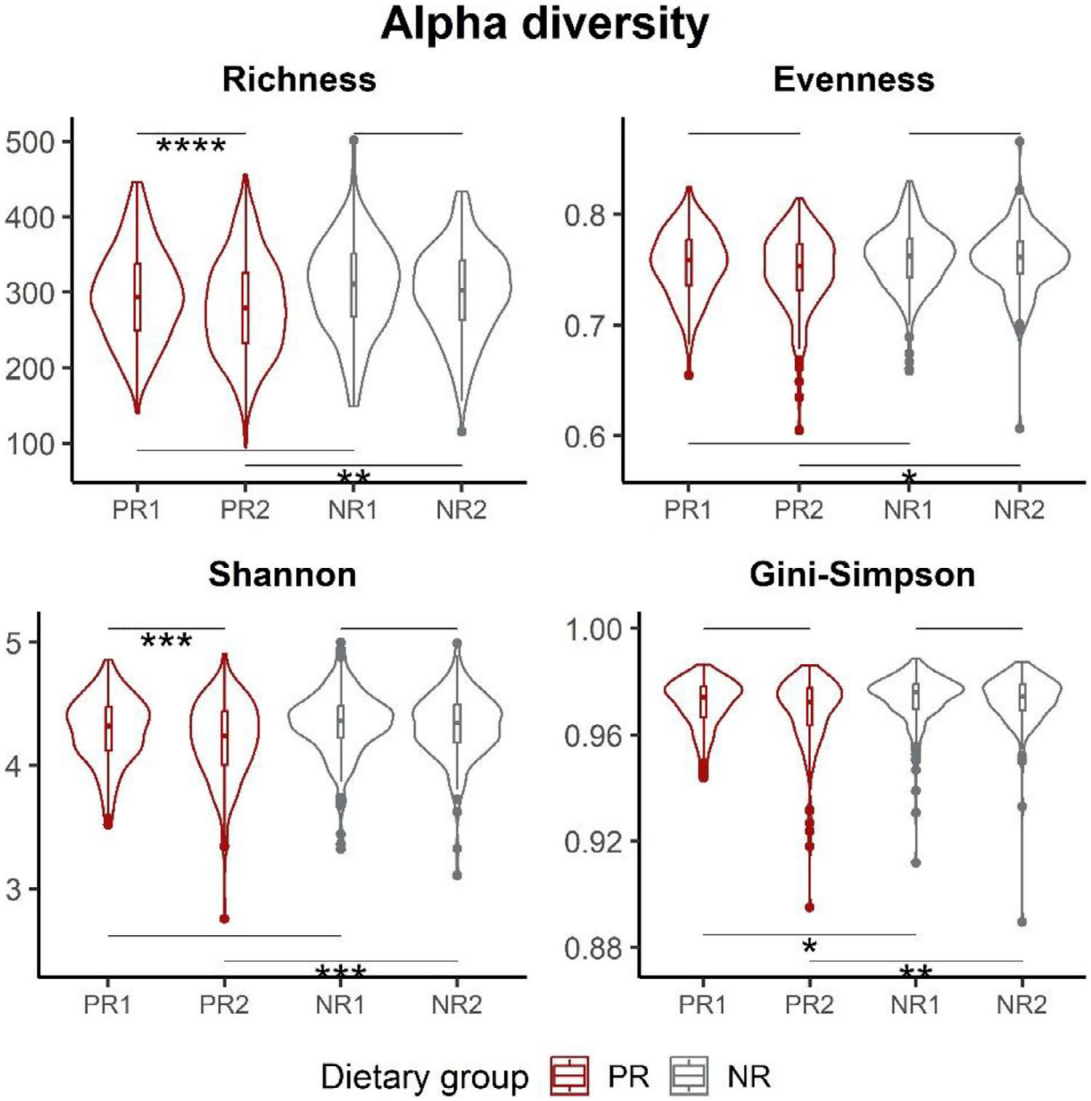
Alpha diversity metrics for each dietary group by time point combination (context). Violin plots and boxplots show the distribution of Observed richness, Pielou's evenness, and Shannon and Gini‐Simpson's diversity indices. The lower and upper hinges of the boxplots correspond to the first and third quartiles. Statistical comparisons were performed using the Wilcoxon signed‐rank test between paired samples (PR1‐PR2, *n* = 186; NR1‐NR2, *n* = 192) and the Wilcoxon rank‐sum test between independent samples (PR1‐NR1, PR2‐NR2). Asterisks indicate levels of significance (*p < 0.05, **p < 0.01, ***p < 0.001, ****p < 0.0001). PR1: PR at T1, PR2: PR at T2, NR1: NR at T1, NR2: NR at T2.

The gut microbiomes of participants were predominantly composed of bacteria from Lachnospiraceae, Ruminococcaceae, and Bacteroidaceae families, and *Bacteroides*, *Faecalibacterium*, and *Blautia* genera (quantified as average abundance per sample; Figure S1A). While the relative abundances of the 20 most prevalent genera were broadly similar across groups and time points (Figure S1B), as expected we found substantial variability between individual participants (Figure S1C) [9]. Short‐term animal product restriction was associated with a reduction in microbial diversity as measured by observed richness and the Shannon diversity index (Wilcoxon, *p* < 0.05), while Pielou's evenness and the Gini‐Simpson index remained unchanged (Wilcoxon, *p* > 0.05) (Figure 2). These findings suggest that animal product restriction, which involves reduced dietary diversity, may primarily lead to loss of low‐abundance taxa. In the control group, microbial diversity remained stable between T1 and T2 (Wilcoxon, *p* > 0.05; Figure 2).

We also explored differences between dietary groups and found that when both groups were omnivorous (T1), microbial diversity was largely comparable, with the exception of lower Gini‐Simpson diversity in the PR group (Wilcoxon, *p* < 0.05; Figure 2), possibly reflecting subtle differences in a few dominant taxa that likely do not affect overall composition and balance. However, during dietary restriction (T2), the PR group exhibited substantially lower microbial diversity across all tested indices compared to the control group (Wilcoxon, *p* < 0.05; Figure 2). Microbial community structure also differed by dietary group and state, as determined by PERMANOVA on Bray‐Curtis distances (R^2^ = 0.0063, q < 0.05) and was additionally associated with age^2^, sex, BMI, Bristol score, medication use, and smoking (0.0019 ≤ R^2^ ≤ 0.0058, q < 0.05; Figure S2).

Finally, to explore the effect of antimicrobial drug use on diversity levels, we conducted a sensitivity analysis by excluding participants who were administered this type of medication between time points. We found minimal differences compared to analyses conducted in the complete set of participants (Text S2 and Figure S3A,B).

### 2.3 Effects of Animal Product Restriction on Bacterial Abundance and Predicted Metabolic Pathways

**FIGURE 3:**
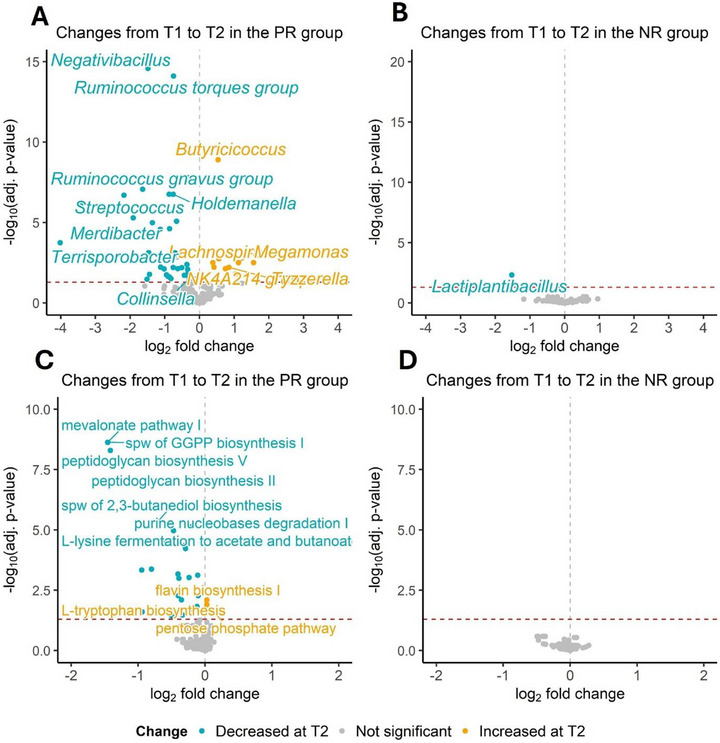
Differentially abundant genera and pathways across time points. Volcano plots displaying log_2_ fold changes and ‐log_10_ p‐values (BH‐adjusted) for 161 bacterial genera tested between time points in the PR group (A) and in the NR group (B), and for 302 microbially expressed predicted pathways between time points in the PR group (C) and in the NR group (D). To detect differentially abundant genera we employed a compound Poisson linear model, while for pathway‐level differential abundance analyses we used a linear model. Significant changes (*p* < 0.05) are highlighted in blue (decreased at T2) or yellow (increased at T2) color. Spw: superpathway.

Dietary restriction altered the relative abundance of nearly one‐third of bacterial genera tested (47 out of 161), while in the control group only *Lactiplantibacillus* was differentially abundant at T2 (Figure 3A,B, Table S2). Three quarters of diet‐associated changes involved a decrease in abundance, with the direction of change being largely consistent within taxonomic families (Table S3). Notably, lactic acid bacteria (LAB) exhibited consistent reductions in abundance, driven chiefly by changes in Lactobacillaceae (*Lactiplantibacillus*, *Lacticaseibacillus*, *Lactobacillus*, *Latilactobacillus*), but also in *Enterococcus*, *Lactococcus*, *Streptococcus*. LAB are commonly derived from fermented dairy products and include strains with probiotic properties [20, 21]. Changes were also found for SCFA‐producing genera, but with variable direction (e.g. increased *Butyricicoccus* and *NK4A214 group*, but decreased *Holdemania* and *Blautia*). Overall, we found that the most pronounced shifts occurred in less abundant families, while dominant families such as Bacteroidaceae, Prevotellaceae, and Bifidobacteriaceae remained largely stable in response to dietary restriction.

We next inferred the functional potential of diet‐responsive shifts in microbial composition and found that 9% (27 of 302) of predicted pathways were affected, with nearly all changes involving downregulation of microbial functions (Figure 3C, Table S2). No changes were found in the control group (Figure 3D; Table S2). Among the predicted pathways, those most strongly downregulated were involved in cholesterol biosynthesis (mevalonate pathway I, superpathway of GGPP biosynthesis I (via mevalonate)), a nutrient that is substantially depleted during dietary restriction of animal products. Downregulation was also found for predicted pathways involved in bacterial cell wall synthesis (peptidoglycan biosynthesis V and II), likely reflecting a reduction in bacterial biomass in accordance with the overall decrease in microbial abundance. Additionally, purine degradation inferred pathways were downregulated (purine nucleobases degradation I, guanosine nucleotides degradation III, purine nucleotides degradation II, adenosine nucleotides degradation II and IV), possibly reflecting a microbial response toward nitrogen conservation under animal product restriction. In contrast, a small number of predicted pathways were upregulated, including flavin biosynthesis I and L‐tryptophan biosynthesis, involved in the production of essential nutrients (vitamin B2 and tryptophan, respectively) that are primarily obtained from animal‐derived foods [22, 23]. The pentose phosphate pathway (PPP), which generates NADPH and ribose‐5‐phosphate for anabolic metabolism and nucleotide synthesis [24], was also upregulated following short‐term restriction.

When comparing compositional and functional profiles between dietary groups, we found that profiles were identical when both groups consumed an omnivorous diet, with the exception of slightly higher abundance of *Faecalitalea* in the PR group. However, three‐to‐four weeks of animal product restriction resulted in divergence of microbiomes between dietary groups, with differences found for 14 genera and 12 metabolic pathways (Figure S4A–D and Table S2). The majority of these differences reflected lower abundance or downregulation in the PR group. Notably, over half of these differences were also detected in the PR group from T1 to T2, suggesting direct effects of dietary restriction on microbial composition and function.

Consistent with the findings for microbial diversity, exclusion of participants who received antimicrobial medication between time points had only a minor impact on differential abundance results, primarily affecting taxa with borderline significance (Text S2, Figure S5A–C and Table S2).

### 2.4 Correlations of Bacterial Abundance and of Metabolic Pathways With Clinical Health Markers

**FIGURE 4:**
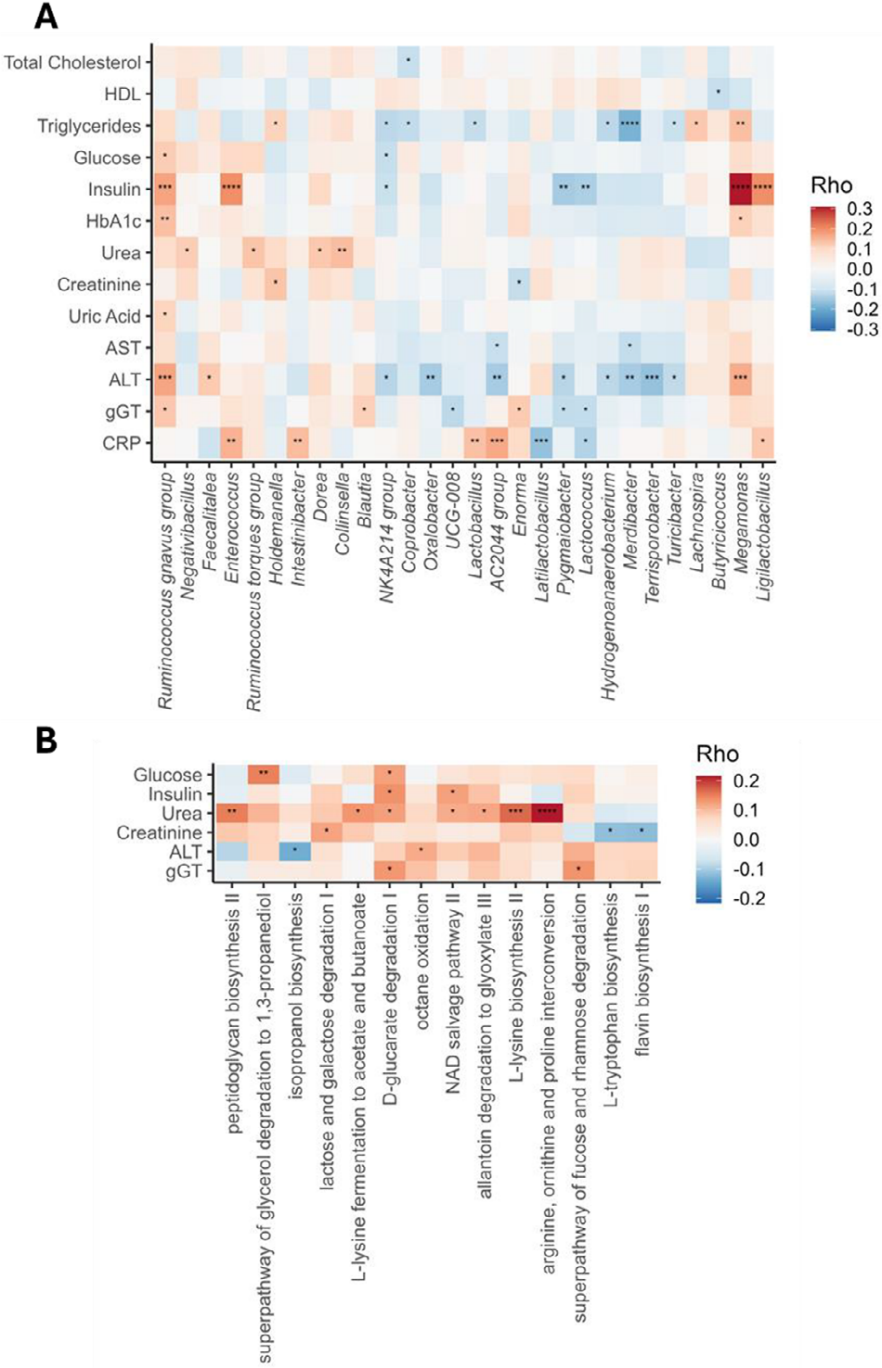
Correlations of bacterial genera with clinical markers of health (blood biomarkers). Heatmaps showing Spearman's correlations of levels of blood biomarkers with bacterial abundance (A) and with expression of predicted metabolic pathways (B). We tested 16 blood biomarkers against 47 and 203 diet‐responsive, differentially abundant genera and predicted pathways respectively. We display biomarkers, genera and pathways with at least one significant correlation. P‐values were BH‐adjusted and asterisks indicate levels of significance (*p < 0.05, **p < 0.01, ***p < 0.001, ****p < 0.0001).

We next examined associations between genera influenced by dietary restriction and blood biomarkers (Figure 4A). A pattern spanning a wide range of bacterial taxonomic groups, was found for correlations of changes in bacterial abundance with levels of triglycerides, insulin, urea, ALT and CRP. The genera underlying most of the correlations were *Ruminococcus gnavus group* (Lachnospiraceae), *NK4A214 group* (Oscillospiraceae) and *Megamonas* (Selenomonadaceae). Similarly, changes in predicted microbial pathways correlated with health markers, including levels of glucose, insulin, urea, creatinine, ALT, and gGT (Figure 4B). Notably, half of these correlations were found for urea, which was mostly linked to carbohydrate or amino acid metabolism pathways.

### 2.5 Multi‐Omics Integration of Microbiome, Metabolomic, and Proteomic Data

**FIGURE 5:**
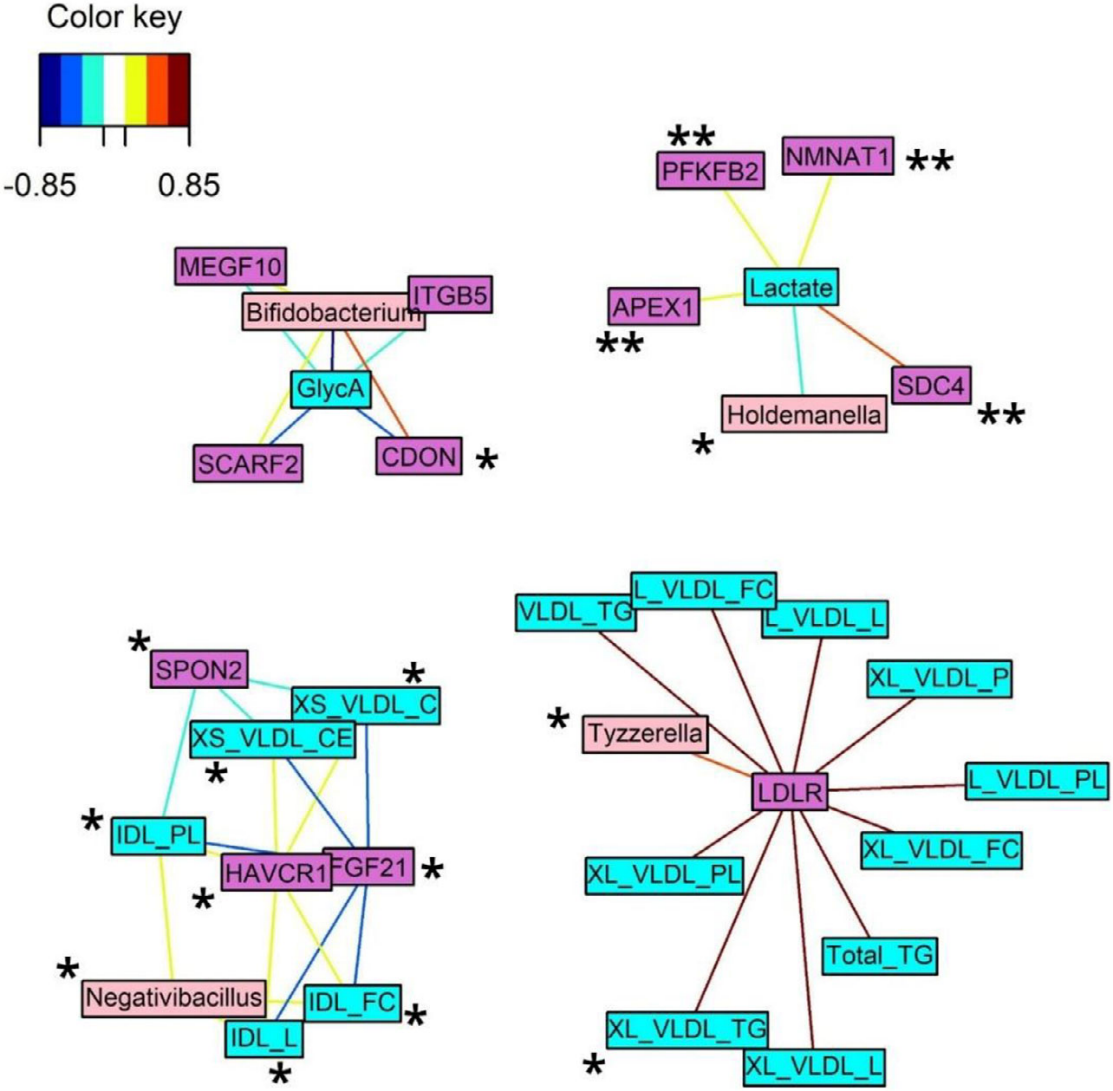
Correlation clusters between gut bacterial genera and host plasma metabolites and proteins. Integration of 161 bacterial genera with 167 plasma metabolites (absolute levels) and 1455 plasma proteins was performed with DIABLO. Clusters encompassing at least one of each biological entities (bacterial genera, metabolites, proteins) and displaying a correlation of |r| ≥ 0.6 were visualized. Light pink nodes represent genera, blue represent metabolites and dark pink represent proteins. Single asterisks indicate entities with changes in abundance in the PR group from T1 to T2. Double asterisks indicate entities with changes in abundance in both the PR and the NR group from T1 to T2. Correlation values are indicated with a color spectrum from blue (−0.85) to red (0.85).

To investigate potential connections between gut microbiome composition and plasma biology, we integrated microbiome, metabolomic and proteomic data and identified four bacterial‐protein‐metabolite correlation clusters (Figure 5; Figure S6). The first cluster was diet‐responsive, consisting of entities that independently exhibited marked changes in response to animal product restriction. *Negativibacillus*, the genus showing the most substantial diet‐responsive decrease in abundance, was associated with FGF21, a protein that increased 2.4‐fold in abundance, as well as with HAVCR1 and SPON2, both among the most diet‐responsive proteins, and with IDL and XS VLDL, lipoprotein subclasses which were markedly decreased during dietary restriction [18].

The second and third clusters included bacterial genera influenced by diet and associated with proteins and metabolites showing mixed responses. *Holdemanella* was correlated with lactate, and with proteins APEX1, NMNAT1, SDC4, PFKFB2, which are primarily involved in glycolysis and redox regulation. *Tyzzerella* was associated with LDLR, and with L VLDL and XL VLDL particles, indicating a potential link with cholesterol metabolism. Components of the fourth cluster did not display diet‐responsive effects and included *Bifidobacterium*, which correlated with GlycA, SCARF2, CDON, ITGB5 and MEGF10, molecules primarily related to inflammatory processes.

## 3 Discussion

In the present study we performed gut microbiome profiling and integrated findings with host plasma biology in a unique group of apparently healthy individuals who follow a consistent and predictable dietary pattern, alternating between omnivory and restriction of animal products for approximately 180–200 days annually. We demonstrated rapid compositional and functional adaptation of the microbiome in response to animal product restriction, and identified correlations between specific bacterial genera, clinical health biomarkers, and host molecular phenotypes. By profiling apparently healthy individuals who follow a naturalistic, highly structured, and prolonged dietary pattern, our findings provide insight into microbiome responses stemming from a real‐life eating behavior. Moreover, integrating microbiome, metabolomic, and proteomic data within a dietary pattern framework provides a systems‐level perspective on diet‐responsive biological pathways relevant to human health, offering valuable insights to guide hypothesis generation for future mechanistic studies.

We found that short‐term restriction of animal products led to a rapid reduction in microbial diversity, without affecting community evenness, likely driven by the selective loss of rare taxa. Although reduced diversity is often linked to disease state and is frequently found in diseases of affluence possibly due to the disruption of complex trophic networks [25], recent research suggests that current diversity metrics may not consistently predict host health status. This arises from the fact that these metrics are not accurate predictors of microbiome function, which has a more recognized influence on health [26]. Meta‐analyses of gut microbiome diversity and composition in healthy and diseased individuals have revealed that although microbial taxonomic composition differs between healthy and diseased individuals, relationships between alpha‐diversity and disease are inconsistent, with the exception of diarrheal diseases [27, 28]. The first studies that focused specifically on the comparison of the human gut microbiome composition across different dietary behaviors reported mixed results [29]. However, more recent research in larger and better‐characterized populations has demonstrated reduced diversity in individuals adhering to plant‐based diets compared to omnivores [10, 30, 31]. Furthermore, plant‐based diets and their associated microbial signatures were linked to more favorable glucose metabolism‐related parameters and cardiometabolic health [10, 31]. We therefore suggest that the reduction in microbiome diversity detected in the present study may reflect a shift in microbiome composition driven by the consumption of a less diverse diet during animal product restriction [10]. During this response, the gut microbiome may shift to align with altered nutrient availability, through the loss of mostly rare taxa. Rare taxa constitute a reservoir of genetic potential, capable of supporting essential microbial functions under diverse conditions [32]. Therefore, their depletion under animal product restriction may reflect a response that may not necessarily be detrimental for health.

Although mostly rare taxa were lost following animal product restriction, we found that the abundance of nearly one‐third of bacterial genera tested was altered, with most changes involving decreased bacterial levels. Genera typically associated with meat and dairy consumption including *Ruminococcus torques group*, *Ruminococcus gnavus group* and *Collinsella*, decreased, while genera commonly linked to plant‐based diets, including *Lachnospira* and *Butyricicoccus*, increased [10, 33, 34, 35]. However, certain fiber‐fermenting genera frequently enriched in vegan diets, such as *Bifidobacterium*, *Anaerostipes* and *Roseburia* [10, 13, 36], remained unaffected. It is plausible that this is because animal product restriction during Lent is typically not accompanied by changes in dietary intake of fiber [16, 17]. Nevertheless, changes in other fiber‐fermenting genera were found, suggesting that the dynamics of these genera may be shaped not only by fiber availability, but also by factors such as microbial competition and ecological niche adaptation [37].

In addition to loss of rare taxa, a marked reduction was found in genera belonging to the LAB group, likely reflecting the loss of dairy exposure during dietary restriction, given that the primary source of these bacteria are fermented dairy products [20]. Indeed, certain LAB taxa, including *Streptococcus*, *Lactobacillus*, *Lacticaseibacillus*, *Lactococcus*, have been shown to be more abundant in omnivorous and vegetarian diets compared to vegan diets and are consistently associated with dairy intake [10, 38, 39], although *Lacticaseibacillus* and *Streptococcus* have also been found to be enriched in individuals following high‐protein diets [13]. As many LAB strains are considered probiotic due to their capacity to produce antioxidant, anti‐inflammatory and immunomodulatory metabolites, this diet‐induced decrease may influence host biology [40, 41].

We identified additional diet‐associated shifts in bacterial abundance for genera that have been linked to various health outcomes in the literature. *Negativibacillus*, which was markedly reduced under dietary restriction, has been associated with inflammatory bowel disease (IBD) [42], non‐alcoholic fatty liver disease [43] and treatment resistance in Crohn's disease [44]. *Merdibacter*, associated with chronic kidney disease (CKD) [45], IBD [46] and irritable bowel syndrome [47], and *Terrisporobacter*, associated with dyslipidemia [48], also showed reduced abundance. *Megamonas*, a genus increasing in abundance under dietary restriction, has been associated with metabolic syndrome [49] and CKD [50]. However, to date, considerable inconsistency exists in the literature regarding the health impact of changes in bacterial abundance for most genera, partly due to the limited resolution of 16S rRNA sequencing. Furthermore, it is often unclear whether changes in microbial abundance are a cause or consequence of disease, further underscoring the challenge of defining a universal ‘healthy microbiome’ signature [51, 52]. Accordingly, diet‐induced changes found in this study cannot be directly linked to health outcomes, but instead highlight specific bacterial candidates for further mechanistic investigation to understand their role in health and disease.

Overall, we found two broad types of inferred microbiome metabolic responses to dietary change, which we refer to as passive and compensatory. The most pronounced passive response was the downregulation of the mevalonate pathway, which plays a key role in the synthesis of isoprenoids and sterols, including cholesterol. During animal product restriction, dietary intake of cholesterol is substantially reduced, a shift also reflected in decreased plasma levels of cholesterol and other lipid classes [18]. In the host, such a reduction typically leads to a compensatory response involving upregulation of the mevalonate pathway and of GGPP biosynthesis, to increase endogenous synthesis of cholesterol and cellular uptake [53, 54]. Consistent with this, our previous work showed that mevalonate kinase, a key enzyme in this pathway [55], increases in plasma abundance following dietary restriction [18]. In the gut microbiome, the mevalonate pathway is primarily expressed by gram‐positive genera, such as *Enterococcus*, *Lactobacillus*, *Streptococcus* (all LAB taxa, that decreased during restriction) as well as *Staphylococcus*, while most gram‐negative bacteria rely on the alternative methylerythritol phosphate (MEP) pathway to synthesize isoprenoids [56]. Thus, it is plausible that this reflects a passive response likely stemming from a nutrient‐driven reduction of genera which employ this pathway.

We also identified compensatory responses of the microbiome to replenish nutrients depleted during animal product restriction. Most notably, we found upregulation of microbial biosynthetic pathways for tryptophan and vitamin B2 (riboflavin), both of which are essential nutrients primarily obtained from animal‐derived foods [22, 23]. Tryptophan serves as a precursor for key molecules such as serotonin, melatonin and niacin (vitamin B3), and plays a central role in the gut‐brain axis, immune regulation, and energy metabolism [22]. Similarly, vitamin B2 is essential for energy metabolism, fatty acid oxidation, purine catabolism, redox balance, and for the metabolism of other B vitamins [23]. Additionally, we detected downregulation of purine degradation, and upregulation of the PPP, which produces ribulose‐5‐phosphate. This shift may also be linked to the upregulation of vitamin B2 biosynthesis, as its production requires both guanosine triphosphate (GTP) and ribulose‐5‐phosphate. These changes in predicted functional potential suggest a microbial response to sustain key metabolic functions and nutrient availability under dietary restriction.

When exploring associations between the microbiome and health‐related biomarkers, we identified correlations between diet‐responsive genera and clinical markers, including triglycerides, insulin, urea, ALT, and CRP. Although diet‐driven microbiome shifts were associated with changes in biomarkers associated with both positive and negative effects on health, we found a consistent link with improved markers of renal function, primarily through decreased levels of urea. This aligns with lower intake of dietary protein during the restriction period [57] and may be reflected in the predicted downregulation of purine degradation pathways during animal product restriction, suggesting a potential microbial response toward nitrogen conservation.

We next explored associations between the gut microbiome and host plasma biology in an integrative analysis bringing together highly correlated features across phenotypes. This approach led to the identification of novel and robust associations between specific bacterial genera and host molecular phenotypes highlighting four distinct clusters, that could provide insight for future mechanistic studies. The first cluster emerged as a diet‐responsive module comprising components that showed the most pronounced changes during dietary restriction. *Negativibacillus*, the genus with the greatest diet‐associated decrease, is a recently discovered taxon [58] whose elevated levels have been associated with resistance to Crohn's disease treatment [44], IBD [42], and non‐alcoholic fatty liver disease [43].

*Negativibacillus* correlated with IDL and XS VLDL lipoproteins, as well as with FGF21, HAVCR1 and SPON2 proteins [18]. VLDL particles, secreted by the liver, transport triglycerides and cholesterol and are progressively converted into IDL through triglyceride depletion [59]. The XS VLDL subclass, having lost much of its triglyceride content, is similar in size and density to IDL, suggesting that this cluster may also capture effects related to lipoprotein particle size. Both VLDL and IDL are components of remnant cholesterol, an atherogenic lipid fraction associated with increased risk of CVD, including coronary heart disease, stroke, cardiac death, and all‐cause mortality [60]. Of the protein components of this cluster, liver‐secreted FGF21 is a hormone with roles in energy homeostasis, lipid and glucose metabolism and insulin sensitivity, and has been shown to extend lifespan in model organisms [61]. In mice, dietary protein restriction induces hepatic FGF21 expression, elevating circulating FGF21 levels in a response shown to be mediated by the gut microbiome [62]. HACVR1 is involved in regulation of immune cell activity and renal regeneration [63], while SPON2 is involved in macrophage activation and has been associated with tumor progression [64], whereas in mice it has been associated with immune response, inflammation and hepatic lipid metabolism [65]. Overall, this cluster appears to capture metabolic adaptations to animal product restriction, and highlights previously unreported connections for further investigation of their likely effects on immunometabolic and cardiometabolic homeostasis.

The other clusters comprised components with mixed responses to dietary restriction, suggesting interactions that are either unresponsive to this dietary shift, or vary in responsiveness across molecular levels. In the second cluster, *Holdemanella* was correlated with lactate, NMNAT1 and APEX1, proteins involved in NAD+ biosynthesis and the oxidative stress response [66, 67], PFKFB2, which mediates lactate‐dependent macrophage glycolysis during efferocytosis in mice [68], and SDC4, which has shown correlations with markers of oxidative stress and inflammation [69]. This cluster likely captures effects of redox regulation, potentially reflecting changes that underlie reduced oxidative stress typical of plant‐based diets [13]. The third cluster included *Tyzzerella*, VLDL particles and LDLR, pointing to a potential link to cholesterol metabolism and cardiovascular health. This is consistent with findings linking *Tyzzerella* to CVD risk [70] and Crohn's disease [71] and with the known role of LDLR in binding VLDL via apoB and apoE [72].

In the fourth cluster, *Bifidobacterium* was associated with GlycA, a marker of systemic inflammation, relevant to CVD risk [73], to CDON and SCARF2, proteins with likely associations to cardiovascular health [74, 75], and to ITGB5, a potential target in treating diabetic cardiovascular complications [76]. Indeed, Bifidobacteria have been shown to reduce levels of TMAO [77], a metabolite typically elevated in meat‐based diets and associated with CVD pathogenesis [8, 11, 78]. While MEGF10, also found in this cluster, has not been directly linked to cardiovascular health, it is a protein likely involved in inflammatory processes through its role in apoptotic cell clearance in the mammalian brain [79]. Although the components of this cluster did not show diet‐responsive effects in their independent analyses, we propose that these correlations may reflect systemic effects of animal protein restriction that could potentially be captured in a larger study group.

Alongside the effects of diet, potential seasonal effects on gut microbiome composition were captured to some extent, by the continuously omnivorous NR group that was profiled during the exact same sampling windows as the PR group (autumn and spring). To date, evidence of seasonal effects on the gut microbiome is mainly from studies of isolated and less industrialized populations, with effects of seasonality being largely attributed to variation in food availability throughout the year [80, 81]. In modern Western lifestyles, a diverse range of foods is available year‐round, likely attenuating these effects. In line with this, our findings suggest minimal seasonal effects on the gut microbiome in this cohort, but the existence of residual effects linked to seasonality cannot be eliminated.

In addition to the above, our study has some key limitations. First, although the design captures the overall impact of animal product restriction, it does not enable us to disentangle effects stemming from specific dietary components, and therefore reflects changes associated with the absence of a broader set of nutrients rather than individual dietary factors. However, this limitation is offset by the value of studying real‐world dietary patterns, which provides more insights than studying individual nutrients in isolation from the broader dietary context [82]. Second, the reliance on 16S rRNA gene sequencing restricts taxonomic resolution to the genus level, and limits functional interpretation to predicted functional potential inferred from taxonomic profiles, rather than direct assessment of microbial gene expression. Given the prominent effects of animal product restriction on gut microbiome composition and function we were nevertheless able to glean broader genus‐level insights, but future metagenomic and metatranscriptomic assays will enable high taxonomic resolution and direct functional profiling. Third, although we have highlighted links between bacteria and blood biomarkers, metabolites and proteins, these represent correlations. The challenge of disentangling causal relationships between microbiome shifts and molecular phenotypes or disease state in humans is widely recognized [83]. To strengthen causal inference, a range of preclinical models, including animals colonized with human faecal microbiota, organoids or organ‐on‐a‐chip systems, is being investigated, although important limitations of these approaches remain [84]. Consequently, further research is needed to establish functional connections and determine causal mechanisms underlying host‐microbiome interactions, thereby enabling a mechanistic understanding of health, and the rational design of targeted therapeutic interventions [84]. Fourth, the metabolomic and proteomic platforms employed covered only a subset of plasma molecules, restricting our analyses to the molecules included in these assays. Finally, although our analyses have been adjusted for confounding factors, we recognize that some residual effects may persist in the results. However, given that our main findings stem from comparisons of the same individual between time points, this means that a lot of these potential effects are to a large extent controlled for.

In conclusion, we have demonstrated that short‐term dietary restriction of animal products induces dynamic remodeling of the gut microbiome. Given the long‐term adherence of participants to this dietary pattern, and considering that both dietary groups exhibited similar diversity, compositional, and inferred functional profiles under omnivorous conditions, the response to dietary restriction appears to be rapid, but it is also most likely transient. While direct causal links between specific genera and health remain challenging to establish, we have highlighted diet‐responsive taxa, including *Negativibacillus*, *Merdibacter*, and *Megamonas*, whose effects remain poorly understood, and suggest that they may serve as candidates for further functional investigation or for biomarker development. Additionally, we revealed correlations between microbial taxa and plasma metabolites and proteins, which can aid in hypothesis generation for causality testing. Overall, our findings suggest potential diet‐responsive effects on health through changes in gut microbiome composition and highlight associations between microbial communities and host physiology. A deeper understanding of how the gut microbiome systemically adapts to dietary changes will shed light on the mechanisms by which diet shapes human health. These insights could inform future strategies for microbiome‐targeted interventions and the development of pharmacological approaches that mimic the beneficial effects of dietary restriction.

## 4 Methods

### 4.1 Population Sample and Study Design

A detailed description of the FastBio (religious Fasting Biology) population sample has been outlined previously [18, 19, 85]. Briefly, participants who met selection criteria (Text S1) belonged to one of two groups, specified by their diet: 200 individuals followed a temporally structured dietary pattern of animal product restriction (periodically restricted, PR group) and 211 continuously omnivorous individuals were not under any kind of special diet (non‐restricted, NR group) (Table S1). The PR diet is practiced for religious reasons and is typically initiated during childhood. It is temporally structured and involves four extended periods of restriction throughout the year, as well as restriction on Wednesdays and Fridays of each week. All participants were invited to two scheduled appointments at the InterBalkan Hospital of Thessaloniki. Appointments were scheduled between 7:30‐9:30 am to minimize circadian effects and were completed during a two‐week window to minimize effects of seasonality. The first appointment (T1 in autumn) was during a period of omnivory for both dietary groups. The second (T2 in spring) took place after three‐to‐four weeks of dietary restriction for the PR group, during Lent (Figure 1A,B).

### 4.2 Collection of Biological Material and Measurement of Traits

Participants collected two stool samples at home up to 48 h prior to their appointment for both sampling time points. Samples were temporarily stored at −20°C and were delivered by participants to the Interbalkan Hospital in an ice pack on the day of their appointment, where material was immediately stored at −80°C. Participants also submitted a completed Bristol score card linked to their sample. Individuals who had taken antibiotic, antifungal, antiviral or antiparasitic medication six months prior to T1 were excluded (Text S1), while participants who had taken this type of medication between T1 and T2 were not excluded from the study, but information on medication use was recorded. The total of FastBio participants were also profiled for 16 blood biomarkers [19], and for 249 and 1455 plasma metabolites and proteins respectively (Nightingale Health Plc, Olink Explore 1536) [18]. Stool samples were delivered by 199 out of 200 PR and all 211 NR individuals. All participants provided written informed consent and the study was approved by the local ethics committee (BSRC Alexander Fleming Bioethics Committee Approval 17/02/2017).

### 4.3 DNA Extraction, Amplification and Sequencing

Microbial DNA was extracted from fecal samples using the QIAamp PowerFecal Pro DNA Kit (QIAGEN), following the manufacturer's protocol. Briefly, 200 mg of fecal material was transferred to a 2 mL bead‐beating tube, and lysis buffer was added. Samples were homogenized for 20 min using a Vortex‐Genie 2 equipped with the QIA.13000‐V1‐24 adapter. Fecal samples were organized in 73 microbial DNA extraction batches in a randomized manner. For each extraction batch, a community standard sample of known composition (ZymoBIOMICS Microbial Community Standard #D6300) was included as a control (Figure S7). DNA purity and concentration were quantified with Nano Drop and Qubit. Library preparation and 16S rRNA sequencing were performed at Oxford Genomics. The V3‐V4 region of the 16S rRNA gene was targeted. Library preparation was performed as described in the Illumina workflow (#15044223 Rev.B). PCR amplicons were purified using Agencourt AMPure XP beads (Beckman Coulter, USA). Indexed amplicons were prepared (Nextera XT Index Kit), pooled in equimolar concentrations, denatured and diluted with a PhiX control and sequenced on a MiSeq system (2 × 300 bp paired‐end reads).

### 4.4 Processing of 16S rRNA Gene Reads and Taxonomic Profiling

Computational analyses were performed in R v4.3.2 [86] and a detailed workflow can be found in Figure S8. Quality control on the sequencing reads was performed with FastQC [87] and MultiQC [88]. Reads were trimmed with Cutadapt [89] to remove sequencing adapters and chimeric PCR constructs. Reads mapping to the human genome (GRCh38) were removed using BWA [90]. Further processing was performed using the DADA2 algorithm [91]. Reads that matched the phiX genome were discarded. An ASV (Amplicon Sequence Variants) table was produced and taxonomy was assigned using the SILVA SSU Ref NR99 v138.1 database [92], formatted for DADA2 [93]. The minimum bootstrap confidence for assignment was set to 50. Assignment as *Incertae sedis* was replaced by NA. We retained only reads with assigned taxonomy at genus level, and samples with data from both time points (paired samples; 188 PR, 194 NR). Each sample yielded an average of 111 483 reads, and a total of 26 855 unique ASVs were retained for downstream analysis.

### 4.5 Quantification of Alpha and Beta Diversity

The ASV table was rarefied (function “rarefy_even_depth”) to control for differential sequencing outputs. The rarefaction threshold was 64 000 reads, resulting in a mean value of 47 892 lost reads per sample, and was chosen to maximize sequencing depth while losing only four individuals (2 PR, 2 NR). Diversity measures were calculated using the R packages “phyloseq” [94] and “vegan” [95] (functions “estimateR”, “diversity”, “ordinate”, “adonis2”). Alpha‐diversity was assessed through four indices which reflect the richness (Observed richness), balance (Pielou's evenness) and diversity (Shannon and Gini‐Simpson indices) of the bacterial community, and the values were compared between the four contexts. For beta‐diversity, the distance matrix was calculated using the Bray‐Curtis dissimilarity index and plotted using principal coordinate analysis (PCoA). We calculated the variance explained by each explanatory variable and used significant factors as covariates in subsequent analyses.

### 4.6 Differential Abundance Analysis of Genera and of Predicted Metabolic Pathways

Using the unrarefied ASV table, we agglomerated ASVs to genus level (function “tax_glom”), filtered for abundance (0.0001%) and prevalence (10%) and rarefied to 60 000 reads. Filtering thresholds were the same for all analyses. We used MaAsLin2 [96] to investigate: a) changes for each dietary group between time points, and b) differences between dietary groups for each time point, taking into account repeated measures and covariates, as described in the Statistical Analysis section and in Text S3.

For functional profiling of the bacterial community, we started with the unrarefied ASV table, performed abundance and prevalence filtering as described above, rarefied to 48 000 reads and employed PICRUSt2 to predict functional abundances [97]. Predicted MetaCyc pathways were subsequently filtered for abundance and prevalence. Changes in pathway expression between contexts were tested using MaAsLin2 (Statistical Analysis and Text S3).

### 4.7 Sensitivity Analysis

To explore whether the use of antibiotic, antifungal, antiviral or antiparasitic drugs affected microbial diversity and abundance, we conducted a sensitivity analysis, using MaAsLin2 in the framework described above, by excluding participants who were administered this type of medication between time points. Of the 33 PR and 35 NR participants belonging to this category, one NR individual was removed after rarefaction due to low number of reads, therefore 33 PR and 34 NR participants were excluded from this analysis.

### 4.8 Associations between Abundance of Bacterial Genera and of Microbial Pathways With Clinical Health Markers

To investigate potential connections between gut microbiome shifts and health‐related blood biomarkers, we explored correlations between diet‐associated changes in: a) bacterial abundance, and b) expression of predicted microbial pathways, with blood biomarker measurements from the same participants from Loizidou et al. [19]. We regressed out effects of covariates (Text S3) and calculated Spearman's correlation coefficients using the “stats” package (function “cor.test”).

### 4.9 Associations between Abundance of Bacterial Genera and Molecular Phenotypes

To explore potential associations between microbiome composition and host plasma biology, we examined correlations between bacterial abundance and plasma metabolite and protein levels. We took into account repeated measures and covariates (Text S3) and used DIABLO [98] (“mixOmics” package [99]) with a full‐weighted design matrix to integrate the three datasets (Text S4). Clusters encompassing at least one of each biological levels (microbiome, metabolites, proteins) and displaying a correlation of |r| ≥ 0.6 were visualized.

### 4.10 Statistical Analysis

Statistical analyses were performed in R v4.3.2 [86]. The 16S dataset comprised 797 samples (T1: 199 PR, 211 NR, T2: 191 PR, 196 NR), all of which were used for taxonomic profiling. Rarefaction and exclusion of samples that did not have data at both time points resulted in a total of 756 paired samples (186 PR, 192 NR) for subsequent analyses. For alpha‐diversity, changes across time points were tested using a Wilcoxon signed‐rank test, and differences between dietary groups using a Wilcoxon rank‐sum test. A *p*‐value < 0.05 was considered statistically significant for all statistical analyses. For beta‐diversity, variance explained by each explanatory variable was calculated with PERMANOVA [100], using a sequential model and 999 permutations, with adjustment for multiple testing. The Benjamini‐Hochberg method was used and an adjusted *p*‐value < 0.05 was considered statistically significant for all statistical analyses involving correction for multiple testing. For genus‐level differential abundance analyses (testing 161 genera), we normalized abundance to the range [0,1] and employed a compound Poisson linear model, while for pathway‐level differential expression analyses (testing 302 predicted pathways) we used a linear model. In both models, for the within‐individual comparisons across time points, participant ID was set as a random effect and time point and selected covariates as fixed effects. The sensitivity analyses, excluding participants who had been administered antimicrobial drugs between T1 and T2, comprised 622 samples (153 PR, 158 NR) and statistical analyses were performed as described above. For the correlation analyses between 161 bacterial genera and 302 predicted pathways with 16 biomarkers, covariate effects were regressed out from each dataset (Text S3) using linear regression modeling. Spearman correlations were computed for each pair of variables and multiple testing adjustment was performed. Finally, for integration of 161 bacterial genera with 167 metabolites (absolute levels) and 1,455 proteins, we added a pseudo‐count of 1 to bacterial abundances and performed centered log‐ratio transformation to mitigate effects of compositionality. Missing metabolite and protein values were imputed with mean value imputation [18]. Covariate effects were regressed out from each dataset (Text S3) using linear regression modeling. To account for repeated measurements, within‐individual variation was extracted using the withinVariation() function from the mixOmics framework [99]. Integration was performed using DIABLO [98]. Detailed information for the configuration of DIABLO can be found in Text S4.

## Author Contributions

Conceptualization: **C.E**. and **A.S.D**. Methodology: **C.E**., **A.S**., **K.R**., and **A.S.D**. Formal analysis: **C.E**. and **K.R**. Investigation: **C.E**., **M.A**., **S.G**., **K.R**., and **A.S.D**. Data curation: **C.E**. Software: **C.E**. Validation: **C.E**. Visualization: **C.E**. Resources: **A.S.D**. Supervision: **N.S**., and **A.S.D**. Writing – original draft: **C.E**. and **A.S.D**. Writing – review and editing: **C.E**., **N.S**., **P.H**., **K.R**., and **A.S.D**. Project administration: **A.S.D**. Funding acquisition: **A.S.D**.

## Funding

This work was funded by an ERC grant to Dr Antigone Dimas (FastBio – 716998).

## Conflicts of Interest

The authors declare no conflicts of interest.

## Supporting information




**Supporting File 1**: advs74549‐sup‐0001‐SuppMat.docx.

**Supporting File 2**: advs74549‐sup‐0002‐TableS2.xlsx

## Data Availability

The data that support the findings of this study are openly available in [European Nucleotide Archive] at [https://www.ebi.ac.uk/ena/browser/view/PRJEB94951], reference number [94951].
