# Supplementary material for: Rapid Remodeling of the Human Gut Microbiome in Response to Short‐Term Animal Product Restriction and Associations with Host Molecular Phenotypes
Source: Adv Sci (Weinh). 2026 Feb 24;13(30):e15575. doi: 10.1002/advs.202515575 (PMC13248852; doi:10.1002/advs.202515575)
Supplement: Supplementary file 1 — Supporting File 1: advs74549‐sup‐0001‐SuppMat.docx. [file ADVS-13-e15575-s001.docx]

Supporting Information

Rapid Remodeling of the Human Gut Microbiome in Response to Short-Term Animal Product Restriction and Associations with Host Molecular Phenotypes

Christina Emmanouil, Maria Anezaki, Alexandros Simistiras, Stavros Glentis, Nikolaos Scarmeas, Pantelis Hatzis, Konstantinos Rouskas, Antigone S. Dimas*

**Text S1.** **FastBio study inclusion and exclusion criteria (from Rouskas et al [1]).**

Inclusion criteria:

- Healthy female and male subjects 18 – 75 years of age at the time of enrolment. One participant turned 76 between time of enrolment and first sampling timepoint (T1).
- Able to provide signed and dated informed consent. Willing to provide blood samples.
- Individuals who had practiced periodic animal product restriction for at least ten years (for the PR group).
- Individuals who had not practiced any kind of specific diet including veganism, vegetarianism, caloric restriction, intermittent fasting (for the NR group).

Exclusion criteria:

- Use of antibiotic, antifungal, antiviral or antiparasitic drugs six months prior to T1.
- Acute disease, defined as the presence of a moderate or severe illness with or without fever, at sampling timepoints.
- Alcohol or drug abuse two years prior to T1.
- Participants who are normally periodically abstaining from animal products, but had to alter their diet for a specific reason (e.g. pregnancy).

**Text S2.** **Sensitivity analyses.**

Between T1 and T2, 33 PR and 35 NR participants were administered antimicrobial medication. To explore whether the use of antimicrobial drugs affected our results, we conducted additional analyses excluding these participants (sensitivity analyses), resulting in a dataset of 622 samples (153 PR, 158 NR). We explored alpha and beta diversity and performed genus-level differential abundance analyses in the same manner as in the analysis conducted on the complete set of participants (main analysis, described in the Statistical Analysis section).

Exclusion of these participants had minimal effects on alpha and beta diversity findings compared to the main analysis. Similarly to the main analysis (**Figure 2**), from T1 to T2, the observed richness and the Shannon diversity index decreased in the PR group, while Pielou’s evenness and the Gini-Simpson index remained unchanged (**Figure S3A**), with only the strength of the significance being affected (Richness in main: p<0.0001, Richness in sensitivity: p<0.01; Shannon in main: p<0.001, Shannon in sensitivity: p<0.01). The PERMANOVA coefficients of determination and adjusted p-values also differed minimally between the two analyses (Main analysis: 0.0019 ≤ R^2^ ≤ 0.0063, 0.0012 ≤ p-adjusted ≤ 0.011; Sensitivity analysis: 0.0022 ≤ R^2^ ≤ 0.0071, 0.0014 ≤ p-adjusted ≤ 0.022) (**Figure S3B)**.

Similarly, exclusion of these individuals had only a minor impact on differential abundance results compared to main analyses. For the comparison between timepoints, the sensitivity analysis in the PR group yielded 41 out of 46 differentially abundant genera detected in the main analysis. Three of the genera not found in the sensitivity analysis narrowly missed the threshold for statistical significance (*Gemella*: p-adjusted = 0.0507, *Turicibacter* and *Hydrogenoanaerobacterium*: p-adjusted = 0.0595) (**Figure S5A**, **Table S2**). In the NR group the sensitivity analysis yielded the same results as the main analysis (**Figure S5A**, **Table S2**). For comparisons between dietary groups, for T1 the sensitivity analysis yielded the same results as the main analysis (**Figure S5B, Table S2**). For T2, the sensitivity analysis yielded 12 out of the 14 genera detected in the main analysis, with the remaining two genera narrowly missing the significance threshold (*Oxalobacter*: p-adjusted = 0.0532, *Gemella*: p-adjusted = 0.0579) (**Figure S5B, Table S2**). Overall, the sensitivity analysis showed high concordance with the main analysis both in terms of statistical significance and effect sizes, as illustrated by the comparison of differential abundance results for the PR group across time points (**Figure S5C**).

**Text S3. Selected covariates.**

For the differential abundance analyses in MaAsLin2, we added sex, age^2^, BMI, medication use, smoking and Bristol score as fixed effects in the LM and CPLM models.

For the correlation analyses between bacterial abundance with levels of blood biomarkers we used linear regression modeling to regress out the effects of:

1. age^2^, sex, BMI, medication use, smoking and Bristol score from bacterial abundance
2. age^2^, sex, BMI, medication use and smoking from biomarker levels

For the correlation analyses between bacterial abundance with molecular phenotypes we used linear regression modeling to regress out the effects of:

1. age^2^, sex, BMI, medication use, smoking and Bristol score from bacterial abundance
2. age2, sex, BMI, medication use and smoking from metabolite levels
3. age2, sex, BMI, medication use, smoking and mean protein value from protein levels

For the correlation analyses between expression of predicted microbial pathways with levels of blood biomarkers we used linear regression modeling to regress out the effects of:

1. age^2^, sex, BMI, medication use and smoking from predicted pathway expression
2. age^2^, sex, BMI, medication use and smoking from biomarker levels

**Text S4: DIABLO configuration.**

Multi-omics integration was performed using DIABLO [2], which employs sparse Partial Least Squares Discriminant Analysis (sPLS-DA) to identify correlations between omics datasets. Matrix factorization is performed through singular value decomposition, and the relationships between the omics datasets are controlled by the design matrix. A full-weighted design matrix was used to maximize the correlation between datasets. Dataset integration was performed using the “block.splsda” function. The maximum number of components to include in the model was set to 10 and the maximum number of iterations of the algorithm for each component was set to 1,000. The optimal number of components was determined using 50x5-fold cross-validation. Classification error rates were calculated using balanced error rates (BER) and the “max.dist” function. The results showed that the error rate decreased monotonically with increasing components, resulting in an overall BER of 0.335 for the 10th component, therefore 10 components were used for downstream analyses. The optimal number of features per component per dataset was determined using 5x10-fold cross-validation through the tune.block.splsda() function. Classification error rates were calculated using BER and the “max.dist” function. The optimal number of features for each component across blocks were:

Bacteria: 30, 25, 5, 5, 25, 20, 20, 20, 15, 15

Metabolites: 5, 5, 5, 5, 5, 5, 10, 5, 5, 10

Proteins: 15, 5, 30, 20, 15, 5, 5, 20, 5, 5

**
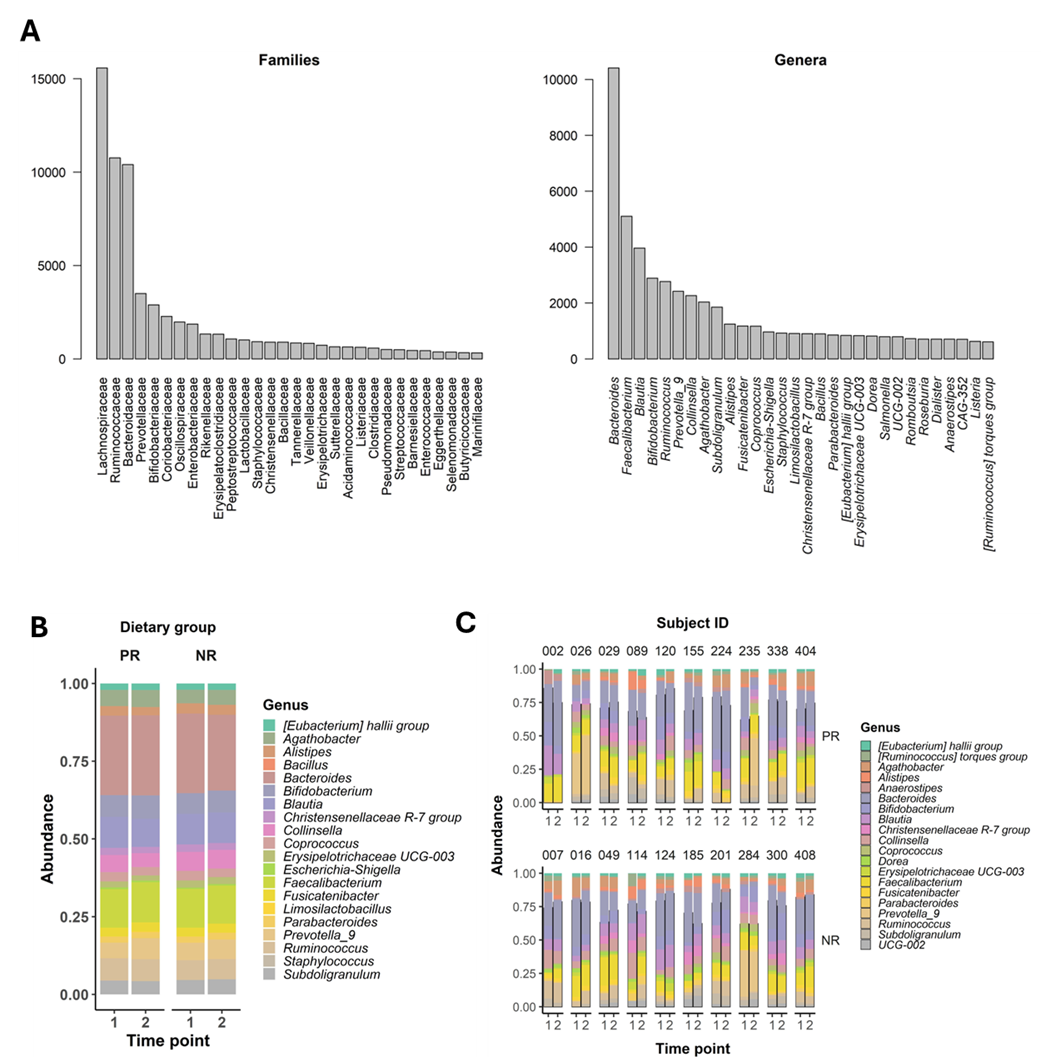
**

**Figure S1. Taxonomic profiling. A**. Bar plots showing the average abundance per sample of the 30 most dominant families and genera. **B, C**. Stacked bar plots showing the relative abundance of the 20 most abundant genera, in all individuals grouped by dietary group and time point (**B**) and in ten participants from each dietary group during each time point (**C**).


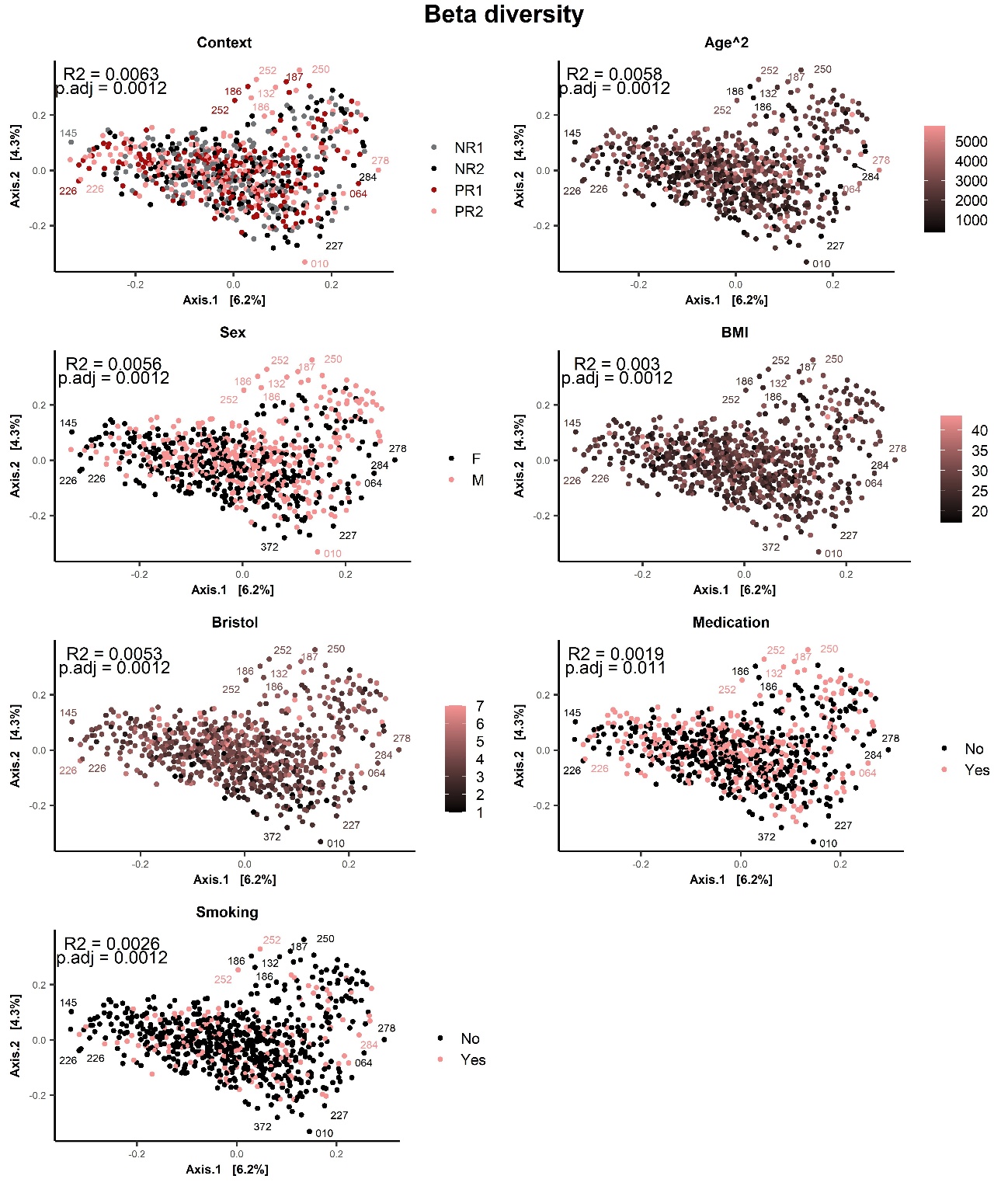


**Figure S2. Beta diversity.** Principal Coordinates Analysis (PCoA) based on Bray-Curtis dissimilarity, showing differences in bacterial composition between samples (n=756). Each dot represents one sample and is colored according to the value of each explanatory variable. Statistical significance of group separation was assessed with PERMANOVA using a sequential model. The coefficients of determination (R^2^) and p-values (BH-adjusted) for 999 permutations are shown.

**
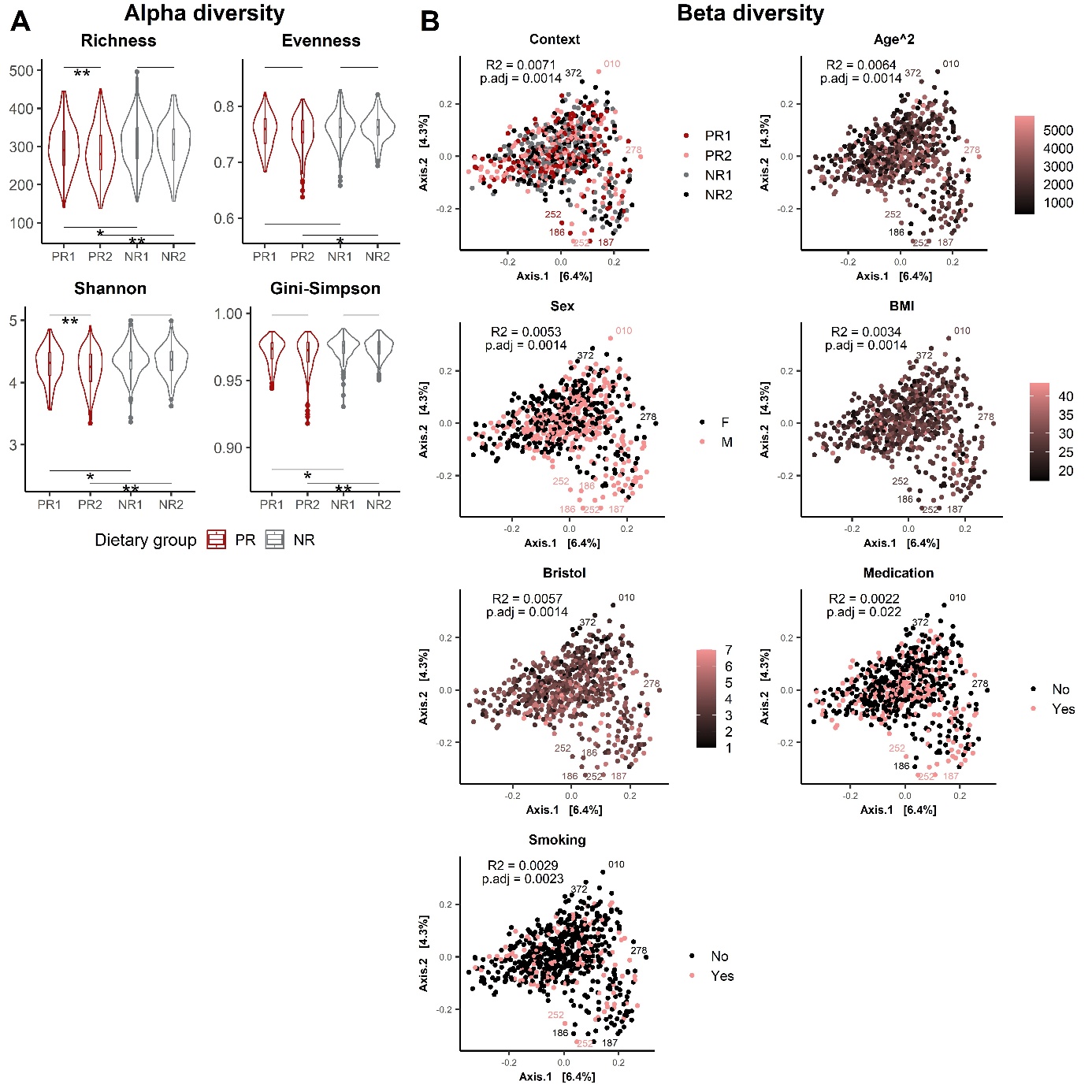
**

**Figure S3. Alpha and beta diversity metrics after exclusion of participants who had been prescribed antimicrobial drugs between time points (sensitivity analyses). A.** Alpha diversity metrics for each dietary group by time point combination. Violin plots and boxplots show the distribution of Observed richness, Pielou's evenness, and Shannon and Gini-Simpson’s diversity indices. The lower and upper hinges of the boxplots correspond to the first and third quartiles. Statistical comparisons were performed using the Wilcoxon signed-rank test between paired samples (PR1-PR2, n=153; NR1-NR2, n=158) and the Wilcoxon rank-sum test between independent samples (PR1-NR1, PR2-NR2). Asterisks indicate levels of significance (*p<0.05, **p<0.01, ***p<0.001, ****p<0.0001). PR1: PR at T1, PR2: PR at T2, NR1: NR at T1, NR2: NR at T2. **B.** Beta diversity. Principal Coordinates Analysis (PCoA) based on Bray-Curtis dissimilarity. Each dot represents one sample (n=622) and is colored according to the value of each explanatory variable. Statistical significance of group separation was assessed with PERMANOVA using a sequential model. The coefficients of determination (R^2^) and p-values (BH-adjusted) for 999 permutations are shown.


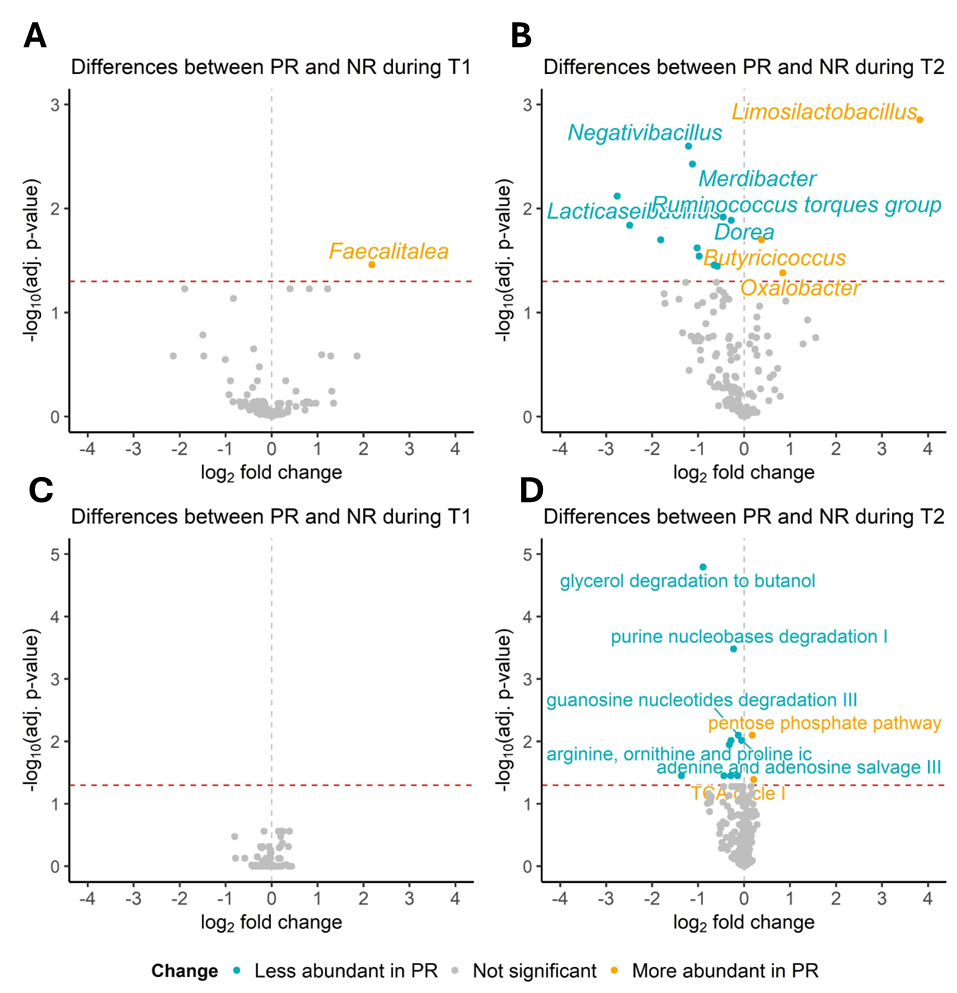


**Figure S4. Differentially abundant genera and pathways between dietary groups.** Volcano plots displaying log_2_ fold changes and -log_10_ p-values (BH-adjusted) for 161 bacterial genera tested between dietary groups at time point 1 (**A**) and at time point 2 (**B**), and for 302 microbially expressed predicted pathways between dietary groups at time point 1 (**C**) and at time point 2 (**D**). For genus-level analyses we employed a compound Poisson linear model, while for pathway-level analyses we used a linear model. Significant changes (p<0.05) are highlighted in blue (less abundant in PR) or yellow (more abundant in PR) color. Ic: interconversion.


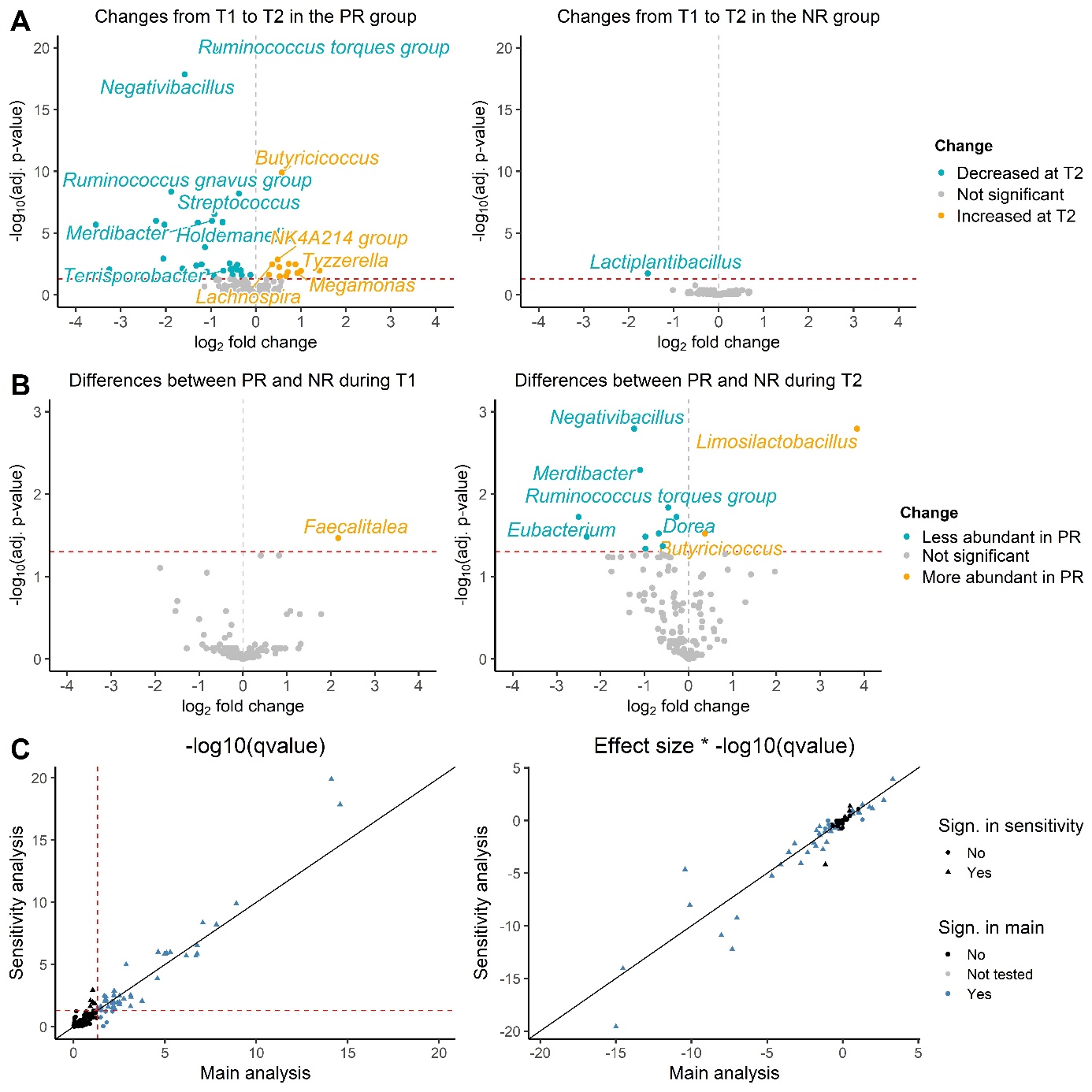


**Figure S5. Differential abundance results after exclusion of participants who had been prescribed antimicrobial drugs between time points (sensitivity analyses). A.** Differentially abundant genera across time points. Volcano plots displaying log_2_ fold changes and -log_10_ p-values (BH-adjusted) for 162 bacterial genera tested between time points in each dietary group. Significant changes (p<0.05) are highlighted in blue (decreased at T2) or yellow (increased at T2) color. **B.** Differentially abundant genera between dietary groups. Volcano plots displaying log_2_ fold changes and -log_10_ p-values (BH-adjusted) for 162 bacterial genera tested between dietary groups at each time point. Significant changes (p<0.05) are highlighted in blue (less abundant in PR) or yellow (more abundant in PR) color. **C.** Comparison of differential abundance results from the main analysis (x axis) and the sensitivity analysis (y axis) for the PR group across time points. -log_10_(q-values) are shown on the left and the products of effect sizes and -log_10_(q-values) are shown on the right. Colors represent significance in the main analysis (significant highlighted in blue) and shapes represent significance in the sensitivity analysis (significant highlighted in triangle). The red lines represent the significance threshold (q<0.05).


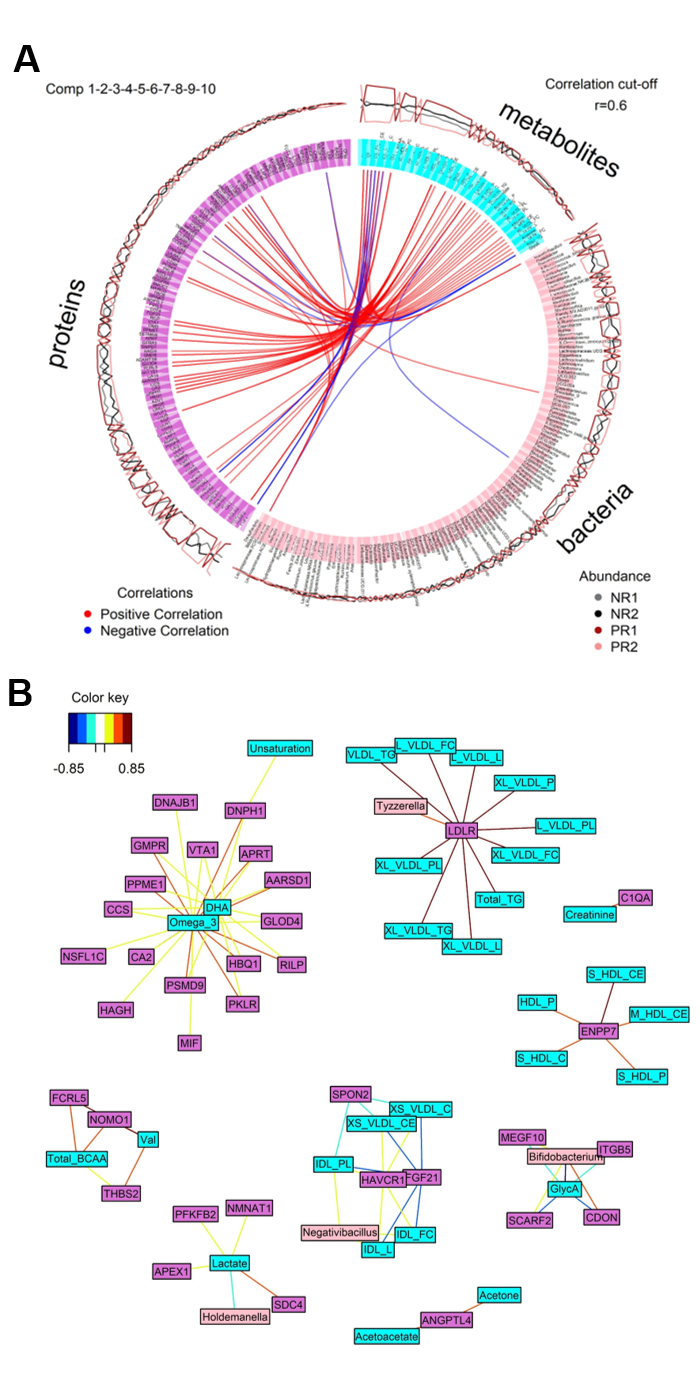


**Figure S6. Correlations between the microbiome, metabolome and proteome datasets.** Integration of 161 bacterial genera with 167 plasma metabolites (absolute levels) and 1,455 plasma proteins was performed with DIABLO. **A.** Circos plot indicating the connectivity between all three datasets, including correlations |r|>0.6. Blue lines indicate negative correlations, red lines indicate positive correlations. The four lines at the outer side of the circle indicate the abundance of each bacterium or molecule in each of the four contexts. PR1: PR at T1, PR2: PR at T2, NR1: NR at T1, NR2: NR at T2. **B.** Network showing all correlations between bacteria, metabolites and proteins included in the circos plot in a simplified manner. Light pink nodes represent genera, blue represent metabolites and dark pink represent proteins. Clusters displaying a correlation of |r|≥0.6 were visualized. Correlation values are indicated with a color spectrum from blue (-0.85) to red (0.85).


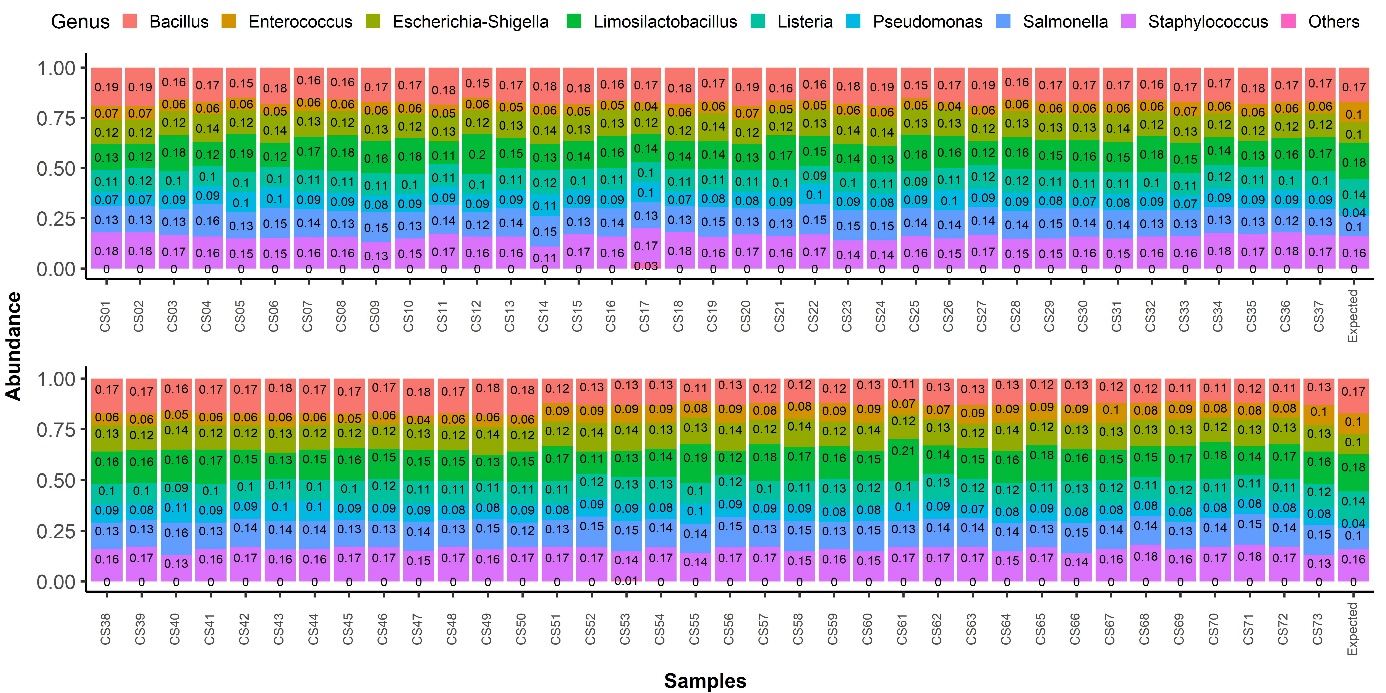


**Figure S7. Assessment of microbial DNA extraction efficiency for each of 73 extraction batches.** A control sample of known composition (ZymoBIOMICS community standard) was included in each of the 73 extraction batches. The theoretical composition for each control sample is 17.4% *Bacillus subtilis*, 9.9% *Enterococcus faecalis*, 10.1% *Escherichia coli*, 18.4% *Lactobacillus fermentum*, 14.1% *Listeria monocytogenes*, 4.2% *Pseudomonas aeruginosa*, 10.4% *Salmonella enterica*, 15.5% *Staphylococcus aureus* and <0.01% alien taxa.


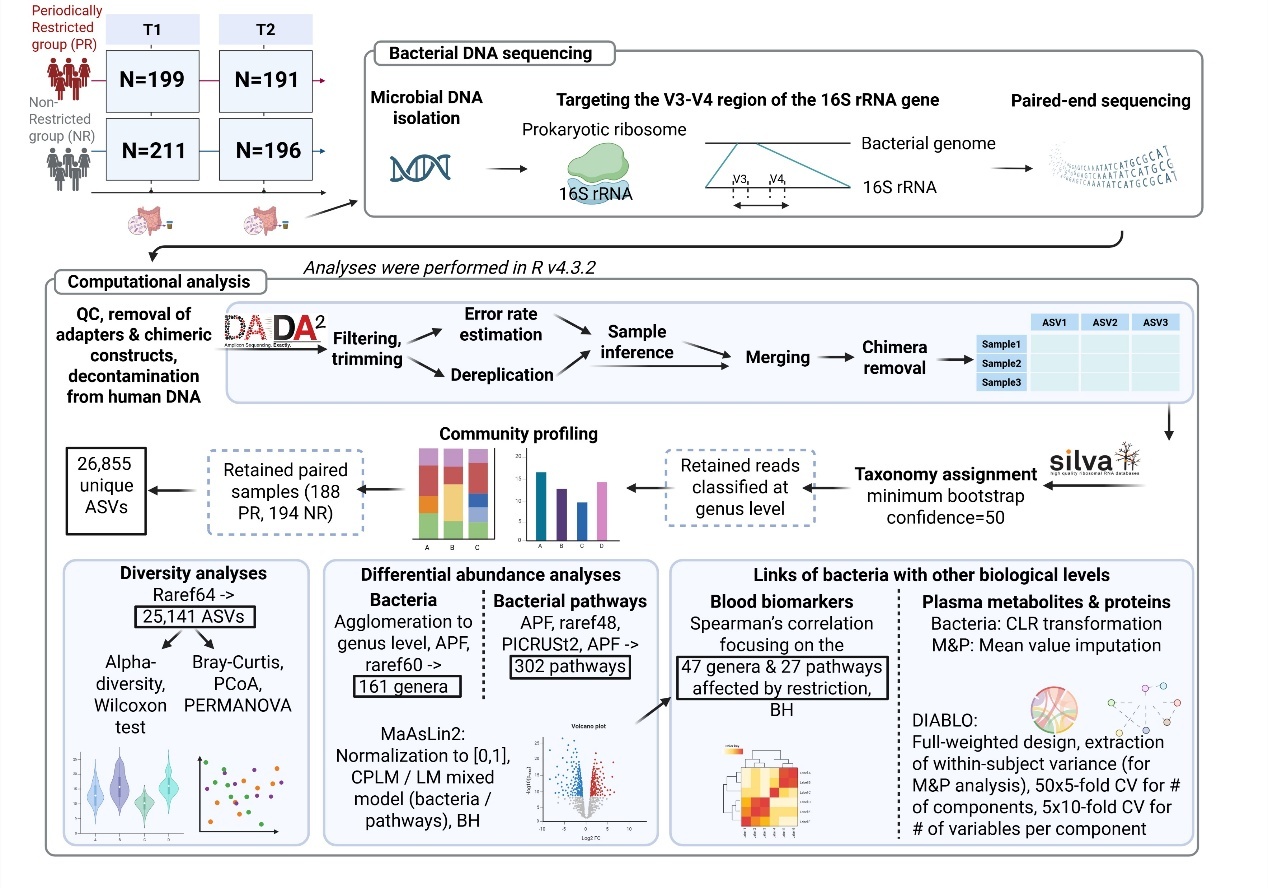
 **Figure S8. Analysis workflow.** APF: abundance (0.0001%) and prevalence (10%) filtering; RarefN: Rarefaction to N thousand reads; BH: Benjamini-Hochberg method for multiple testing correction; M&P: Metabolites and proteins. All significance thresholds are equal to 0.05. Created in BioRender. Emmanouil, C. (2026) https://BioRender.com/k85f93a

**Table S1. Sociodemographic traits and geographic origin of FastBio study participants.** The FastBio population sample has been described in detail in Rouskas et al [1]. Continuous variables were expressed as mean ± standard deviations and categorical variables as N (%). SBP = Systolic Blood Pressure; DBP = Diastolic Blood Pressure. P-values are from chi-square test or Mann-Whitney test for categorical and numerical variables, respectively. Stool samples were delivered by 199 PR and 211 NR individuals.

|  | **Total (N=411)** | **PR (N=200)** | **NR (N=211)** | ***P*-value** |
| --- | --- | --- | --- | --- |
| **Sex** |  |  |  |  |
| Female | 224 (54.5) | 108 (54) | 116 (55) | 0.8425 |
| Male | 187 (45.5) | 92 (46) | 95 (45) |  |
| **Age (yrs)** | 48.1 ± 13.6 | 51.5 ± 13.5 | 45.0 ± 13.1 | <0.00001 |
| **BMI (Kg/m^2^)** | 27.3 ± 4.6 | 28.4 ± 4.6 | 26.2 ± 4.4 | <0.00001 |
| **Blood Pressure (mmHg)** |  |  |  |  |
| Systolic BP (SBP) | 124 ± 19.4 | 127 ± 19.0 | 121 ± 19.7 | 0.003 |
| Diastolic BP (DBP) | 79 ± 11.3 | 80 ± 10.8 | 78 ± 11.8 | 0.04 |
| **Education** |  |  |  |  |
| Tertiary | 293 (71.3) | 134 (67) | 159 (75.4) | 0.0612 |
| Primary and Secondary | 118 (28.7) | 66 (33) | 52 (24.6) |  |
| **Marital status** |  |  |  |  |
| Married | 286 (69.6) | 147 (73.5) | 139 (65.9) | 0.0931 |
| Unmarried | 125 (30.4) | 53 (26.5) | 72 (34.1) |  |
| **Smoking (Y/N)** |  |  |  |  |
| Non-smokers | 328 (79.8) | 187 (93.5) | 141 (66.8) | <0.00001 |
| Smokers | 83 (20.2) | 13 (6.5) | 70 (33.2) |  |
| **Parental origin** |  |  |  |  |
| Northern Greece | 275 (66.9) | 137 (68.5) | 138 (65.4) | 0.0979 |
| Central or Southern Greece | 32 (7.8) | 11 (5.5) | 21 (9.9) |  |

**Table S2**. **Differentially abundant genera and pathways across contexts.** (Excel file)

**Table S3. Direction of changes in abundance upon dietary restriction for members of families that encompass more than one affected member.**

| **Family** | **Genus** | **Direction** |
| --- | --- | --- |
| Anaerovoracaceae | *Eubacterium nodatum group* |  |
|  | *Family XIII AD3011 group* |  |
| Butyricicoccaceae | *Butyricicoccus* |  |
|  | *UCG-008* |  |
| Coriobacteriaceae | *Enorma* |  |
|  | *Collinsella* |  |
| Erysipelatoclostridiaceae | *UCG-004* |  |
|  | *Erysipelatoclostridium* |  |
| Erysipelotrichaceae | *Holdemanella* |  |
|  | *Holdemania* |  |
|  | *Clostridium innocuum group* |  |
|  | *Merdibacter* |  |
|  | *Faecalitalea* |  |
|  | *Turicibacter* |  |
| Lachnospiraceae | *Ruminococcus torques group* |  |
|  | *Dorea* |  |
|  | *Ruminococcus gnavus group* |  |
|  | *Lachnospira* |  |
|  | *AC2044 group* |  |
|  | *Tyzzerella* |  |
|  | *UCG-010* |  |
|  | *Frisingicoccus* |  |
|  | *Blautia* |  |
| Lactobacillaceae | *Lactiplantibacillus* |  |
|  | *Lacticaseibacillus* |  |
|  | *Lactobacillus* |  |
|  | *Latilactobacillus* |  |
|  | *Ligilactobacillus* |  |
| Oscillospiraceae | *NK4A214 group* |  |
|  | *Intestinimonas* |  |
|  | *Hydrogenoanaerobacterium* |  |
|  | *Flavonifractor* |  |
| Peptostreptococcaceae | *Terrisporobacter* |  |
|  | *Intestinibacter* |  |
| Ruminococcaceae | *Negativibacillus* |  |
|  | *UBA1819* |  |
|  | *Pygmaiobacter* |  |
| Streptococcaceae | *Streptococcus* |  |
|  | *Lactococcus* |  |

**References**

1. Rouskas, K., et al., *Periodic dietary restriction of animal products induces metabolic reprogramming in humans with effects on cardiometabolic health.* npj Metabolic Health and Disease, 2025. **3**(1): p. 14.

2. Singh, A., et al., *DIABLO: an integrative approach for identifying key molecular drivers from multi-omics assays.* Bioinformatics, 2019. **35**(17): p. 3055-3062.
